# Physiology of Methylotrophs Living in the Phyllosphere

**DOI:** 10.3390/microorganisms9040809

**Published:** 2021-04-12

**Authors:** Hiroya Yurimoto, Kosuke Shiraishi, Yasuyoshi Sakai

**Affiliations:** Division of Applied Life Sciences, Graduate School of Agriculture, Kyoto University, Kitashirakawa-Oiwake, Sakyo-ku, Kyoto 606-8502, Japan; yurimoto.hiroya.5m@kyoto-u.ac.jp (H.Y.); shiraishi.kosuke.7t@kyoto-u.ac.jp (K.S.)

**Keywords:** methanol, methylotrophs, phyllosphere, diurnal adaptation, plant growth promotion

## Abstract

Methanol is abundant in the phyllosphere, the surface of the above-ground parts of plants, and its concentration oscillates diurnally. The phyllosphere is one of the major habitats for a group of microorganisms, the so-called methylotrophs, that utilize one-carbon (C1) compounds, such as methanol and methane, as their sole source of carbon and energy. Among phyllospheric microorganisms, methanol-utilizing methylotrophic bacteria, known as pink-pigmented facultative methylotrophs (PPFMs), are the dominant colonizers of the phyllosphere, and some of them have recently been shown to have the ability to promote plant growth and increase crop yield. In addition to PPFMs, methanol-utilizing yeasts can proliferate and survive in the phyllosphere by using unique molecular and cellular mechanisms to adapt to the stressful phyllosphere environment. This review describes our current understanding of the physiology of methylotrophic bacteria and yeasts living in the phyllosphere where they are exposed to diurnal cycles of environmental conditions.

## 1. Introduction

**Figure 1:**
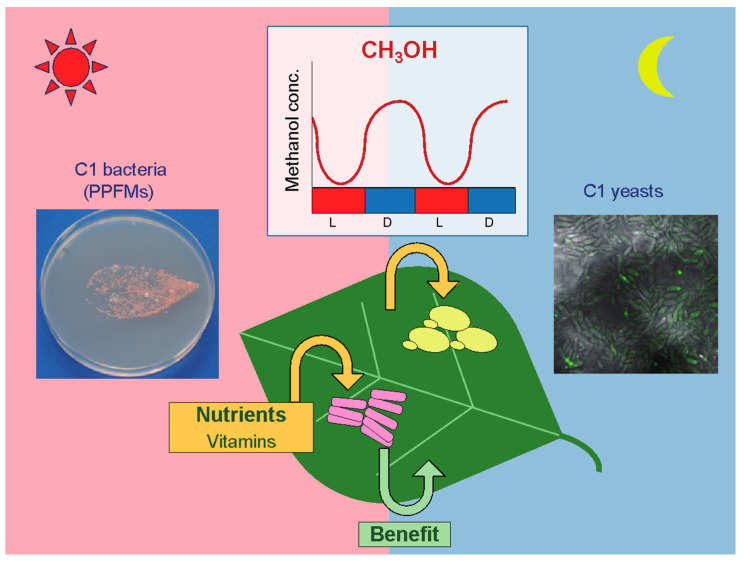
Colonization of methanol-utilizing methylotrophs in the phyllosphere where methanol concentrations oscillate diurnally. Methanol-utilizing bacteria (one-carbon (C1) bacteria, pink-pigmented facultative methylotrophs (PPFMs)) and yeasts (C1 yeasts) colonize the surface of plant leaves and acquire nutrients produced by plants. Concentrations of methanol in the phyllosphere oscillate diurnally, with lower concentrations in the light period (L) and higher concentrations in the dark period (D). After the leaf printing on the agar medium containing methanol as the sole carbon source, pink-pigmented colonies were observed (left panel photo). *Candida boidinii* cells expressing the fluorescent protein Venus proliferate on *Arabidopsis thaliana* leaves (right panel photo).

In nature, methanol is ubiquitous. Its main origin is considered to be the methyl ester groups of pectin, one of the major components of the plant cell wall [1,2]. Methanol is produced through the hydrolysis of pectin methyl esters by pectin methylesterase, and released from the plant stomata. Once released, methanol can be utilized by microorganisms living in the phyllosphere, defined as the aerial parts of plants, or emitted into the atmosphere as a volatile organic compound whose global emission is estimated to be 100 Tg per year [1,3,4]. The atmospheric concentration of methanol has been reported to fluctuate depending on the opening and closing of stomata [5]; however, the amount of methanol in the phyllosphere had not been quantified. Recently, we revealed that the concentration of methanol available for microorganisms on the surface of plant leaves also oscillates during the daily light–dark cycle. Results showed that the methanol concentration in the phyllosphere was higher in the dark period and lower in the light period, which was opposite to atmospheric methanol (Figure 1) [6,7], suggesting that phyllospheric microorganisms utilize the methanol hydrolyzed from the plant pectin in a direct manner, rather than using methanol present in the air.

A wide variety of microorganisms colonize the phyllosphere and the area of soil surrounding plant roots (rhizosphere). Interactions between plants and microbes have been considered to affect not only the growth and proliferation of both organisms, but also the ecosystem and global environment. The total area of the global phyllosphere is estimated to be 10^9^ km^2^, twice as large as the surface of the earth, and such space could be colonized by bacterial populations of 10^26^–10^27^ cells, as well as lower numbers of archaea and fungi [8]. While plant–rhizobia and plant–mycorrhizae interactions in the rhizosphere have been thoroughly investigated, studies of plant–microbe interactions in the phyllosphere have been limited to those involving plant pathogens. As such, positive and neutral interactions between phyllospheric microbes and their host plants have been closely researched only in the last decade [9,10].

Among phyllospheric microorganisms, methanol-utilizing bacteria, known as pink-pigmented facultative methylotrophs (PPFMs), are the dominant colonizers of plant leaf surfaces (Figure 1) [11,12]. Methylotrophs are a diverse group of microorganisms that utilize reduced one-carbon (C1) compounds, such as methanol and methane, as their sole sources of carbon and energy. C1-utilizing microorganisms include bacteria, archaea, and fungi. Most methylotrophic fungi are yeasts. PPFMs are members of the genus *Methylobacterium*, although some have recently been reclassified into *Methylorubrum* [13], and some of these are known to have the ability to promote plant growth [14,15]. Along with PPFMs (C1 bacteria), some methylotrophic yeasts (C1 yeasts), which belong to the genera *Candida* and *Komagataella*, also colonize the phyllosphere (Figure 1) [6]. These yeasts can grow vigorously on methanol-containing media and have been used as hosts for heterologous protein production using strong and regulatable methanol-induced gene promoters [7,16,17,18,19]. Because of their intracellular dynamics, these yeasts have also been used as model organisms to investigate the molecular and cellular mechanisms of the development and degradation peroxisomes, which are essential organelles for methanol metabolism.

In addition to methanol, plants emit methane, which is anaerobically generated by methanogens in soil via their aerenchyma, although some plants aerobically produce methane, which is assumed to be formed during the synthesis of pectin methyl ester groups coupled with photosynthesis [20,21]. Methane-utilizing methylotrophs, known as methanotrophs, also colonize the surface of plants [22,23,24,25,26]. Methane- and methanol-utilizing methylotrophs inhabit various environments in nature besides the phyllosphere, and play important roles in the global carbon circulation of two major greenhouse gases, methane and CO_2_.

Phyllospheric microorganisms are exposed to a variety of environmental factors, such as low nutrients, temperature, drafts, and UV light, which change diurnally [9]. Methylotrophs living in the phyllosphere have therefore evolved physiological adaptations to grow and survive under such stressful conditions. In this review, we summarize our recent studies and current understanding of the physiology of methylotrophs with respect to their dominant colonization and survival on the surface of plant leaves. Finally, we describe our recent results on the improvement of rice crop yields by foliar spraying of PPFM cells in paddy fields.

## 2. Physiology of Methylotrophic Yeasts in the Phyllosphere

### 2.1. Proliferation of Methylotrophic Yeasts on Plant Leaves Where Methanol Concentrations Fluctuate Diurnally

The ability to utilize methanol as a carbon source is considered to be one of the reasons why methylotrophs are the dominant colonizers of the phyllosphere. This hypothesis is supported by the fact that *Methylorubrum extorquens* AM1 mutant strains defective in methanol metabolism are less competitive than the wild-type strain during colonization on plant leaves [27,28].

While methylotrophic yeasts have often been isolated from various plant resources, it was unknown until recently whether these yeasts can proliferate in the phyllosphere. The methylotrophic yeasts *Candida boidinii* and *Komagataella phaffii* (*Pichia pastoris*) were found to proliferate on the leaf surface of growing *Arabidopsis thaliana* plants [6]. Yeast cells expressing a fluorescent protein were inoculated onto plant leaves and their growth was observed by fluorescence microscopy and quantitative PCR analysis for two weeks. We found that *C. boidinii* cells grew slowly, replicating approximately 3−4 times within 11 days of inoculation. Furthermore, *C. boidinii aod1∆* and *das1∆* strains in which genes encoding the peroxisomal methanol-metabolizing enzymes alcohol oxidase (AOD) and dihydroxyacetone synthase (DAS), respectively, were disrupted could not proliferate on leaves, indicating that methanol metabolism is necessary for growth in the phyllosphere.

Another question that had not been answered until recently was how much methanol is present on plant leaves and available to methylotrophs. To examine the methanol concentration in the phyllosphere, we developed a cell-based methanol sensor using the methylotrophic yeast *C. boidinii* expressing the fluorescent protein Venus under the control of the methanol-induced *DAS1* gene promoter [6]. The sensor cells were inoculated on the surface of leaves of *A. thaliana* plants that had been growing for 2–3 weeks after germination in a plant growth chamber with a daily light-dark cycle (14 h light, 10 h dark). After a 4 h incubation, the fluorescence intensity was measured. The estimated methanol concentration was higher in the dark period (25–60 mM) than in the light period (0–5 mM), suggesting that the local methanol concentration in the phyllosphere of growing young leaves oscillates during the daily light-dark cycle (Figure 1). In addition, transcript levels of the methanol-induced genes *AOD1* and *DAS1* corresponded to the phyllospheric methanol concentration measured by the sensor cells. In contrast to young leaves, the methanol concentration on wilting or dead leaves was estimated to be greater than 250 mM, and did not show diurnal oscillation. Given that the amount of methanol available to phyllospheric microorganisms fluctuates naturally, it is reasonable to propose that methanol-induced gene expression in methylotrophic yeasts was acquired through evolution to adapt to the phyllospheric environment.

We also investigated the nitrogen sources utilized by *C. boidinii* in the phyllosphere [29]. Since *C. boidinii* can utilize nitrate and methylamine as nitrogen sources, we focused on *YNR1*, encoding nitrate reductase, and *AMO1*, encoding amine oxidase, and examined their physiological functions in the phyllosphere. The wild-type and *amo1Δ* strains were able to proliferate on growing young leaves of *A. thaliana* plants, whereas the *ynr1Δ* strain could not. The *YNR1* gene, but not the *AMO1* gene, was expressed in cells inoculated on young leaves, and its expression level fluctuated diurnally, indicating that the nitrate concentration fluctuates diurnally. Further observation, however, found that expression of the *AMO1* gene was induced on wilting leaves. These results suggest that available nitrogen sources for *C. boidinii* change from nitrate on young leaves to methylamine on wilting leaves. Subsequently, we investigated the in vitro fate of *YNR1* after alternating the nitrogen source from nitrate to methylamine, and found that a selective autophagic pathway was involved in the nitrate metabolic change. Together, these results indicate that carbon and nitrogen sources available to methylotrophs in the phyllosphere change not only during the day–night cycle, but also during the life cycle of the plant.

### 2.2. Molecular and Cellular Mechanisms of Adaptation to the Phyllosphere Environment in Methylotrophic Yeasts

During growth on methanol, methylotrophic yeasts develop large numbers of peroxisomes that contain AOD, DAS, and other key enzymes for methanol metabolism [17]. When cells are shifted to a glucose or ethanol medium from a methanol medium, peroxisomes are degraded by peroxisome-specific autophagy, which is termed pexophagy. In the phyllosphere environment, where methanol concentrations oscillate diurnally, peroxisome dynamics should be determined by the methanol concentration. We observed that the number of peroxisomes in *C. boidinii* cells on young leaves increased in the dark period and decreased in the light period, corresponding to the methanol concentration [6]. Furthermore, our results demonstrated that *C. boidinii* mutants with disruptions in Pex5 (responsible for peroxisomal protein import), Atg1 (a pivotal kinase for all autophagic pathways), and Atg30 (a receptor molecule on peroxisomes recognized by core Atg proteins) were unable to proliferate on plant leaves, which revealed that regulation of peroxisome dynamics is essential for the proliferation of methylotrophic yeasts in the phyllosphere.

**Figure 2:**
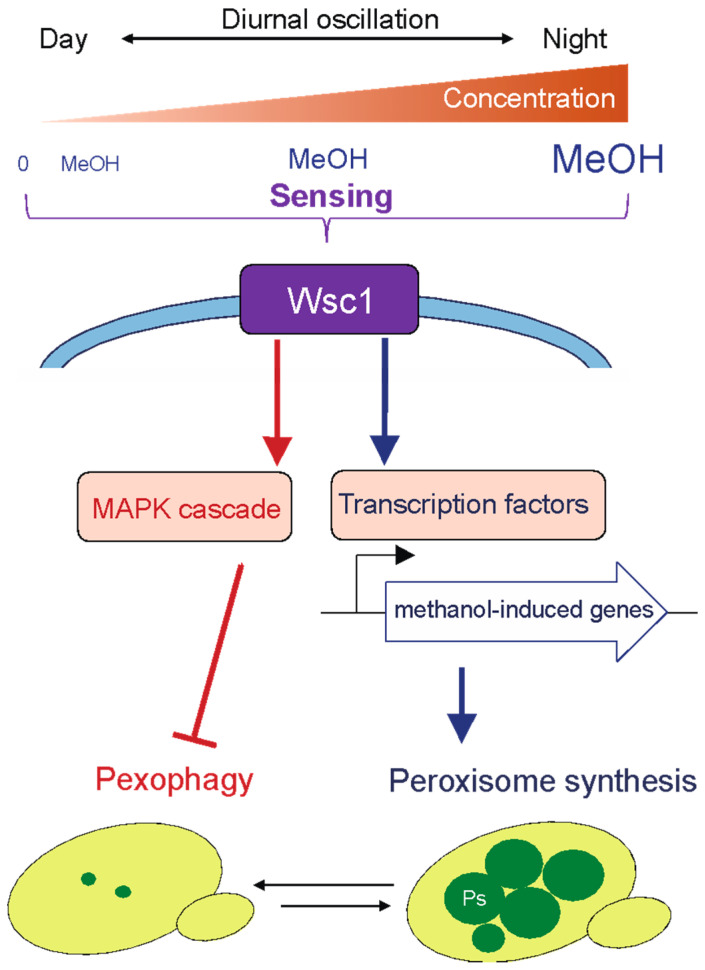
The cell-surface protein Wsc1 senses the methanol concentration in the phyllosphere and regulates peroxisome dynamics in methylotrophic yeast. Wsc1 senses a wide range of methanol concentrations that oscillate diurnally in the phyllosphere. A signal from Wsc1 is transmitted to the transcription factors, activating expression of methanol-induced genes followed by the development of peroxisomes. Under lower methanol concentrations and carbon source-depleted conditions, peroxisomes are degraded by pexophagy. Wsc1 and the downstream MAPK cascade repress pexophagy in the presence of methanol concentrations higher than 0.15%.

To adapt to the phyllosphere environment and regulate cellular functions in response to the methanol concentration, methylotrophic yeasts must be able to sense a wide range of methanol concentrations in the phyllosphere. We found that the cell-surface proteins Wsc1 andWsc3 in *K. phaffii* are responsible for sensing the environmental concentration of methanol and for regulating methanol-induced gene expression, i.e., genes encoding proteins involved in peroxisome synthesis and methanol metabolism [30]. Moreover, KpWsc1 and its downstream MAPK (a mitogen-activated kinase) cascade negatively regulate pexophagy in the presence of methanol (higher than 0.15%) through suppression of Atg30 phosphorylation [31]. These results indicate that Wsc1 regulates not only methanol-induced gene expression followed by the development of peroxisomes, but also pexophagy in response to the methanol concentration sensed by the two distinct signaling pathways (Figure 2).

## 3. Ubiquitous Colonization of PPFMs on the Surface of Plants and Species-Level Specific Interactions

### 3.1. Distribution of Methane- and Methanol-Utilizing Bacteria in the Phyllosphere

Plant leaf surfaces support a large microbial community. The dominant microbial inhabitants are considered to be bacteria, whose population on leaves is estimated to be 10^6^–10^7^ cells/cm^2^ [8]. We have investigated the distribution of methane- and methanol-utilizing bacteria in the phyllosphere and isolated many strains from plant-associated materials [22,23]. In contrast to PPFMs, little was known about the habitation and distribution of methanotrophs in the phyllosphere. Some previous metagenomic and metaproteomic analyses did not detect methanotrophs on plant leaves [32,33], but other studies did in fact detect small populations [12,34]. We set up enrichment cultures with methane to obtain methanotrophs living on plants and demonstrated that methanotrophs could be cultivated from 12% of the phyllosphere samples [22]. Furthermore, we found that both submerged and floating aquatic plants associated with methanotrophs have high methane consumption activity, and revealed that these hydrophytes serve a niche purpose for methanotrophs, functioning together as an important sink of methane [23,35].

Many PPFMs have been isolated from plant-related materials, and the community composition and population size of PPFMs in the phyllosphere have been analyzed with culture-independent approaches [11,12]. A proteogenomics analysis revealed that alphaproteobacterial *Methylobacterium* and *Sphingomonas* are the major genera among phyllospheric bacteria; for example, each genus represented more than 20% of the population on soybean leaves [33].

### 3.2. Species Level Specificity between PPFMs and Plants

We investigated the number of PPFMs (colony forming units (CFU)/g fresh leaves) on various kinds of vegetable leaves planted in a home farm (ca. 100 m^2^) and discovered that the value varied from 10^5^ to 10^7^ CFU/g [36]. These results suggest that the plant species affects the population size of PPFMs on leaves. Furthermore, we found that the red perilla plant (*Perilla frutescens crispa* (Thunb.) Makino) harbored a dominant population of PPFMs, and isolated the representative strain *Methylobacterium* sp. OR01 from red perilla seeds. PPFMs with an identical 16S rRNA gene sequence to *Methylobacterium* sp. OR01 were dominant in almost all PPFM communities of red perilla plants cultivated at four geographically different places in Japan, confirming geographically-independent species-level specific PPFM-perilla plant associations [37]. Thus, the plant species influences the dominant *Methylobacterium* species regardless of geographical and environmental factors.

We also confirmed the direct transmission of *Methylobacterium* sp. OR01 from red perilla seeds to their leaves and the competitiveness of this strain on red perilla plants [37]. We compared the colonization ability of *Methylobacterium* sp. OR01 and *M. extorquens* AM1 on red perilla plants using different antibiotic-resistant strains. Although both strains colonized the red perilla leaves when inoculated separately onto seeds, only *Methylobacterium* sp. OR01 colonized leaves when both strains were inoculated together in a mixed cell suspension. These results indicate that *Methylobacterium* sp. OR01 has a greater ability to colonize red perilla plants than *M. extorquens* AM1.

### 3.3. Pantothenate Auxotrophy of Methylobacterium sp. OR01 and Fitness Advantage in the Phyllosphere Environment

In addition to carbon and nitrogen sources, some trace cofactors such as vitamins can be utilized by microorganisms in the phyllosphere [38]. We found that most PPFMs isolated from living plant samples, including *Methylobacterium* sp. OR01, required pantothenate (vitamin B_5_) for growth on a minimal medium. Since pantothenate is synthesized by the condensation of pantoate and β-alanine in bacteria, we tested whether these compounds could restore the ability of *Methylobacterium* sp. OR01 to grow on a minimal medium. Results showed that the addition of β-alanine and its biosynthetic precursors, spermine, spermidine, 5,6-dihydrouracil, *N*-carbamoyl-β-alanine, and 3-hydroxypropanoate, restored growth, indicating that pantothenate auxotrophy of *Methylobacterium* sp. OR01 occurs due to the absence of the β-alanine biosynthetic pathway.

*Methylobacterium* sp. OR01 could colonize the leaf surface of *A. thaliana* cultivated on a plant medium that did not contain pantothenate or its precursors, and we confirmed that pantothenate, β-alanine, and several precursor compounds were present on the *A. thaliana* leaves [38]. These results suggest that the pantothenate-auxotrophic strain OR01 colonizes the surface of plant leaves by utilizing not only pantothenate, but also β-alanine and some other precursors produced by the host plant (Figure 1), and that the ability of the pantothenate auxotrophic strain to synthesize β-alanine from multiple compounds enabled this strain to adapt to various environments, including the phyllosphere. When *Methylobacterium* sp. OR01 was inoculated on *A. thaliana* seeds with *M. extorquens* AM1, which is able to synthesize pantothenate as well as other B vitamins, the pantothenate-auxotrophic strain OR01 dominated over the nonauxotrophic strain AM1 on the leaves. One possible reason for the auxotrophic strain having greater colonization ability than the nonauxotrophic strain might be attributed to the fact that the auxotrophic strain does not need to consume energy for the synthesis of pantothenate. *Methylobacterium* sp. OR01 might gain increased fitness by acquiring pantothenate and its precursors, which are sufficiently present in the phyllosphere, thus saving the energy costs of the biosynthesis of these compounds.

## 4. Survival Strategy to Adapt to Various Environmental Stresses in the Phyllosphere

### 4.1. General Stress Response Regulator PhyR in Methylotrophs

Phyllosphere microorganisms are exposed to various kinds of environmental stresses, such as extreme temperatures, UV radiation, drafts, osmotic pressure, reactive oxygen species (ROS), and low nutrients, and must therefore adapt to these diurnally changing stresses. Regulating the expression of stress-response genes is one strategy to adapt to such environmental stresses. The general stress response regulator, PhyR, was first identified as an abundantly produced protein in *M. extorquens* AM1 and was found to be involved in plant colonization [39,40,41]. PhyR, which is exclusively found in *Alphaproteobacteria* bacteria, is an anti-anti-sigma factor in a conserved signal cascade consisting of PhyR, NepR, and SigT [42]. The *M. extorquens* AM1 *phyR* mutant strain was not only impaired in phyllosphere colonization, but also demonstrated increased sensitivity to general stresses, such as heat, UV light, osmolarity, and ROS. Since *Alphaproteobacteria* bacteria are dominant in the phyllosphere as mentioned above, the general stress response system regulated by PhyR might contribute to enhanced fitness in phyllosphere environments. We isolated an alphaproteobacterial methanotroph, *Methylosinus* sp. B4S, from a plant leaf and revealed that PhyR in this strain was also involved in resistance to heat shock and UV light [43].

### 4.2. Role of KaiC Family Proteins in M. Extorquens AM1

**Figure 3:**
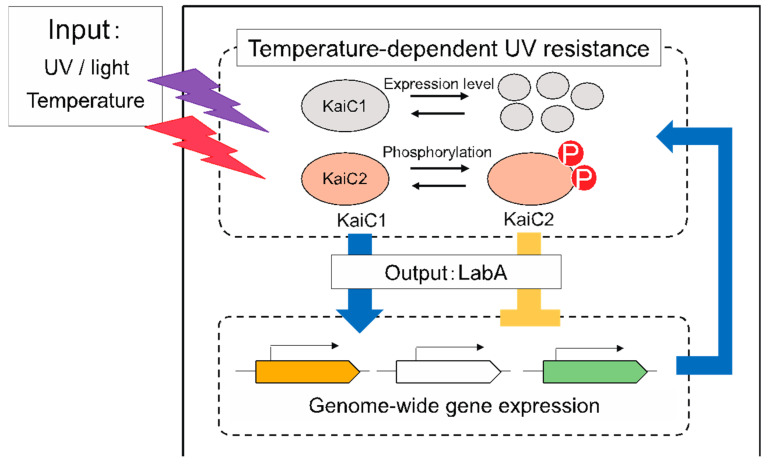
Proposed regulatory network for integrative control of temperature-dependent UV resistance in *M. extorquens* AM1. When cells are exposed to UV light and/or high temperatures, the positive regulator KaiC1 is induced and the negative regulator KaiC2 is phosphorylated. The expression level of KaiC1 and the phosphorylation state of KaiC2 regulate genome-wide gene expression through the downstream regulator LabA. Both the *kaiC1* and *kaiC2* genes are regulated by downstream effectors.

As already stated, phyllosphere microorganisms adapt to various environmental cues affected by the day-night cycle. We have investigated the role of the *M. extorquens* AM1 KaiC proteins, which are homologues of the circadian clock generator components in cyanobacteria, and found that they function to adapt to multiple environmental stresses, namely temperature and UV light in *M. extorquens* AM1 (Figure 3) [44].

KaiC proteins are the central component of the circadian clock system in cyanobacteria and perform both autokinase and autophosphatase functions [45,46]. KaiC phosphorylation is stimulated by KaiA, and KaiB reduces the effects of KaiA. The phosphorylation level of KaiC exhibits an environment-independent oscillation with an approximately 24 h period and the Kai protein complex regulates global gene expression through downstream regulators such as LabA. KaiC family proteins are conserved among many bacterial and archaeal species, including *Methylobacterium* spp. and *Methylorubrum* spp.; however, the physiological role and regulation of phosphorylation of KaiC proteins in noncyanobacterial microorganisms is unclear.

*M. extorquens* AM1 has two *kai* gene clusters, *kaiC1-kaiB2-kaiB1-kaiR1* and *kaiC2-kaiR2*. Both KaiC1 and KaiC2 from strain AM1 have conserved serine residues corresponding to the phosphorylation sites of the cyanobacterial KaiC. We investigated the phosphorylation states of KaiC1 and KaiC2 by immunoblot analysis [44]. Mutations in the serine residues in KaiC1 (S432A and S433A, KaiC1m) and KaiC2 (S426A, KaiC2m) resulted in the disappearance of the phosphorylated bands, indicating that both KaiC1 and KaiC2 are phosphorylated on these conserved residues.

In order to study the importance of the *kaiC* genes and the *labA* gene for plant colonization by *M. extorquens* AM1, competitive colonization tests between the wild-type and gene-disrupted strains were conducted on *A. thaliana* plants. Colonization by the *∆kaiC2*, *∆kaiC1∆kaiC2*, and *∆labA* strains was significantly lower than that of the wild-type strain, indicating that KaiC2 and LabA are necessary for optimal colonization of strain AM1 in the phyllosphere. In addition, since the phosphorylation-defective mutant KaiC2m was unable to restore the colonization ability of the *∆kaiC2* strain, the conserved serine residue at position 426 in KaiC2 and its phosphoregulation seem to be necessary for plant colonization.

Further analyses revealed that the *∆kaiC1* strain was more sensitive to UV light than the wild-type strain, but both *∆kaiC2* and *∆labA* strains were more resistant to UV light and high temperatures than the wild-type strain. These results suggest that KaiC1 and KaiC2 have opposing regulatory functions. When the wild-type strain was exposed to UV stress at different temperatures, the survival ratio of the wild-type strain after UV treatment increased with increasing growth temperatures (24–32 °C). Thus, *M. extorquens* AM1 exhibited temperature-dependent UV resistance (TDR). At all tested temperatures (24 °C, 28 °C, and 32 °C), the *∆kaiC1* strain had lower viability than the wild-type strain under UV stress conditions. It is interesting to note that while the *∆kaiC2* strain had a higher survival ratio than the wild-type strain at 24 °C and 28 °C, the viability of the two strains was comparable at 32 °C. The *∆kaiC1∆kaiC2* strain exhibited an intermediate phenotype between those of the *∆kaiC1* and *∆kaiC2* strains. We therefore concluded that KaiC1 and KaiC2 function as positive and negative regulators, respectively, in the TDR phenotype. Based on analyses of KaiC1 and KaiC2 protein levels and their phosphorylation status at different temperatures, we found that the amount of KaiC1 protein and the phosphorylation level of KaiC2 decreased with increasing growth temperatures. These results indicate that the amount of KaiC proteins and the phosphorylation state of KaiC2 control the UV resistance pathway in an integrated manner according to the growth temperature, thus allowing cells to adapt to changing environmental conditions (Figure 3).

## 5. Improvement of Crop Yield by PPFMs

Some PPFMs have the ability to promote plant growth through the production of phytohormones, such as auxins and cytokinins, and induce systemic resistance against pathogens and diseases [14]. Additionally, PPFMs have some functions that may improve plant nutrition, such as siderophore production, phosphate solubilization, and N_2_ fixation. Since *Methylobacterium* spp. and *Methylorubrum* spp. can be cultivated to very high cell densities using methanol as a carbon source [47], it is easy to prepare large amounts of cells for use in the field. Thus, the use of PPFMs as plant biostimulants might contribute to the methanol bioeconomy, in which a variety of useful compounds are biologically produced from natural gas- or biomass-derived methanol.

Scattered reports have demonstrated the promotion of plant seedling growth and an increase of total plant biomass following treatment with PPFMs (via seed inoculation or foliar spraying) under laboratory conditions or pot-scale cultivation, particularly for vegetables [48,49,50,51]. However, improvement of crop yields at the field level has not been well investigated. We recently reported improved rice crop yields following foliar spraying of PPFMs in a commercial paddy field [52]. After selection of PPFM strains and rice (*Oryza sativa*) cultivars based on the results of in vitro seedling growth tests, we conducted paddy field experiments. Our results demonstrated that the crop yield of the sake-brewing rice cultivar Hakutsurunishiki was reproducibly improved in a commercial paddy field for over a five-year period by foliar spraying of PPFMs. We tested and optimized the timing of PPFM inoculation and found that foliar spraying of not only living cells but also killed cells or a cell wall polysaccharide fraction improved rice crop yields. Furthermore, we found that a one-time foliar spray of PPFM after the heading date was effective in increasing the rate of ripening and crop yield. Although the underlying mechanism regarding how PPFMs or their components act after the heading date of rice is unclear, our results suggest that the positive effect is due to a direct stimulation by PPFM cell wall components during the translocation stage of rice growth.

## 6. Future Perspectives

The phyllosphere environment is quite different from the rhizosphere for microorganisms and is affected by diurnal changes of light, temperature, and nutrients derived from plant metabolism, including photosynthesis. In order to elucidate the mechanism of interactions between plants and phyllosphere microorganisms that significantly influence the global ecosystem, it is necessary to deepen our understanding of the phyllosphere environment and the physiology of both plants and microorganisms. In this paper, we described our current understanding of the physiology of methylotrophs that colonize the phyllosphere. Methanol-utilizing bacteria and yeasts were revealed to have diverse metabolic and physiological functions that allow them to adapt to and dominate in the phyllosphere environment. Another key discovery was that there is species-level specificity between PPFMs and plants not only in dominant colonization in the phyllosphere, but also for improving crop yields. Further studies will focus on the principles of the symbiotic interactions between methylotrophs and plants at the molecular level, particularly by examining the molecular basis of species-level specificity and the mechanism of colonization dominance. In addition, since PPFM cells can be cultivated at high-cell density with methanol, which can be derived from methane or renewable biomass, application of PPFMs to agriculture has the potential to increase the input of natural gas-derived carbon atoms into biomass. The practical use of the synergistic interactions between methylotrophs and plants should facilitate explorations in agriculture and in environmental technology.

## Data Availability

Not applicable.
